# Descended marginal sinus of the placenta

**DOI:** 10.1002/ccr3.2092

**Published:** 2019-03-18

**Authors:** Eiji Ryo, Shigenari Namai, Ippei Nakagawa, Ranka Kanda, Keita Yatsuki, Takuya Ayabe

**Affiliations:** ^1^ Department of Obstetrics and Gynecology, School of Medicine Teikyo University Tokyo Japan

**Keywords:** descended marginal sinus, low‐lying placentation, marginal sinus of the placenta, placenta previa, placental edge, rupture of the marginal sinus

## Abstract

This is the first reported case of descent of the placental marginal sinus through the cervix to the external os. We think marginal sinus rupture does exist. The definition of placental edge should be the parenchyma in diagnosis of low‐lying placentation. Clinically, however, the low‐lying marginal sinus should be treated similar to low‐lying placentation.

## 1 INTRODUCTION

This is the first reported case of descent of the placental marginal sinus through the cervix to the external os.

For decades, limited attention has been paid to the marginal sinus of the placenta. A Pub Med reference search with the keywords “placenta” and “marginal sinus” only revealed 43 titles, of which approximately two‐thirds were published in the 1950s and 1960s.

However, with the widespread use of transvaginal ultrasonography, there have been growing opportunities to observe the echo‐free region with slow blood flow located at the lower margin of the placenta. This region is termed as the marginal sinus of the placenta.

Here, we report a pregnant woman who presented with unusual images related to a low‐lying placenta. In this case, the marginal sinus of the placenta descended through the opened cervix to the external os. This is the first reported case of a descended marginal sinus. Based on this case, we discussed the existence of marginal sinus rupture and the definition of low‐lying placentation.

## 2 CASE PRESENTATION

**Figure 1:**
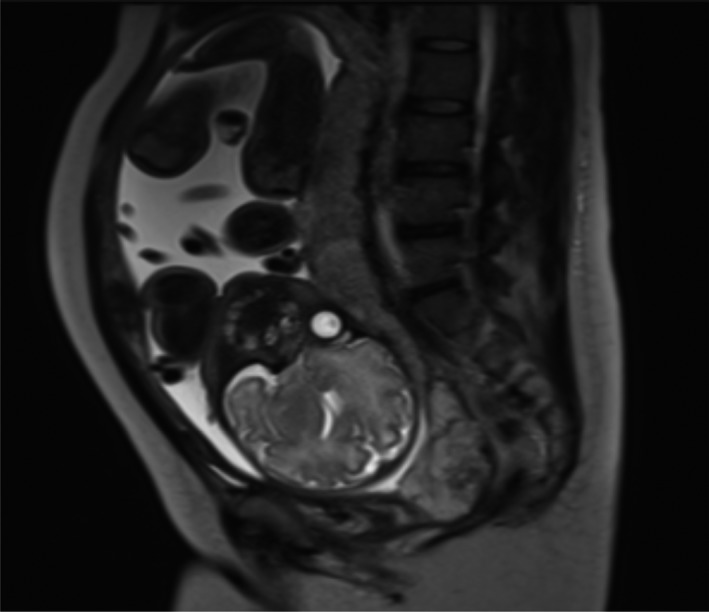
T2‐weighted sagittal MRI. Abnormality is demonstrated at the cervical area

A 38‐year‐old woman, gravid 0, underwent freeze‐thaw embryo transfer due to a male sterility complication. She became pregnant and had no complications other than a 4‐cm diameter fibroid in the anterior wall of the uterus. Her pregnancy progressed uneventfully though posterior wall low‐lying placentation, but not placenta previa was noted. She underwent routine examinations at 33 weeks of gestation, and transvaginal ultrasonography revealed abnormal blood flow at the cervical region. She underwent MRI (Figure 1), which also revealed abnormal findings. She was estimated to have a high risk of massive obstetric hemorrhage though a precise diagnosis could not be obtained. She was transferred to the maternal‐fetal intensive care unit in our hospital.

**Figure 2:**
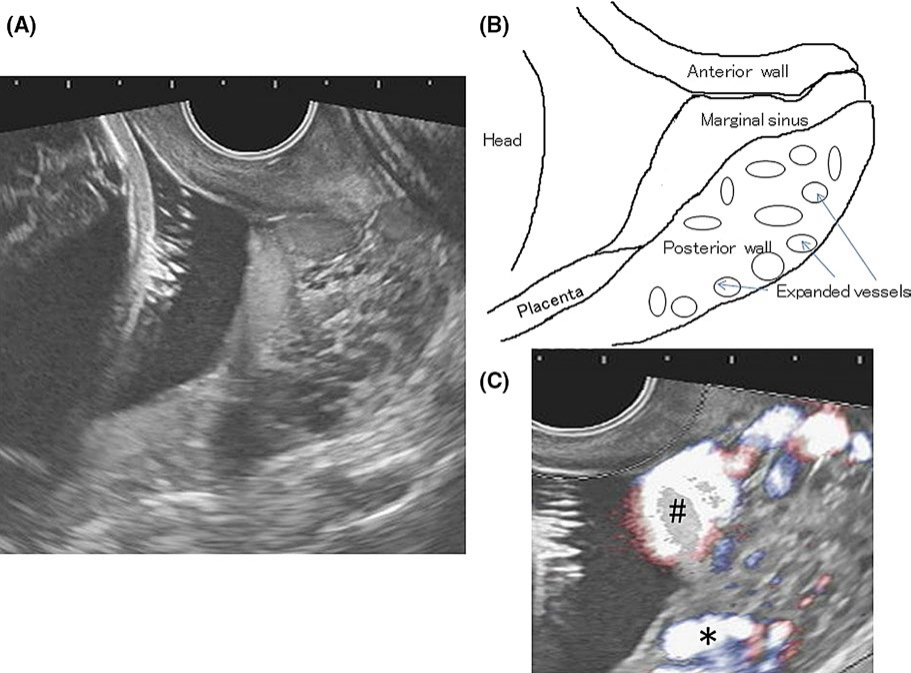
Transvaginal ultrasonography. A, Transvaginal sonography of the cervical area (B‐mode). B, Schema explaining A. The cervix is open. The placental parenchyma is low lying. The placental marginal sinus descends through the opened cervix to the external os. The vessels at the posterior cervix are expanded. C, Color‐image. (#) Blood flow within the marginal sinus. (*) Blood flow within the expanded vessel at the posterior cervix. There is no blood flow connection between the two

Radiologists in our hospital interpreted the MRI as either invasive placentation into the cervix or cervical vascular anomalies, though they had never seen images like these. An expert opinion of the transvaginal ultrasonography finding (Figure 2) was as follows. The cervix was open. Placental parenchyma was low lying but not covering the internal os. The placental marginal sinus seemed to descend through the opened cervix to the external os. Vessels at the posterior cervix wall were enlarged, and no direct connection was found between the vessels and the descended marginal sinus.

The woman and her family consented to Cesarean hysterectomy if necessary. Late preterm cesarean delivery was performed at 36 weeks and 2 days of gestation to avoid emergent cesarean hysterectomy in case of invasive placentation with massive bleeding. A male neonate weighing 2774 g was delivered. Apgar scores were 7 (1 minutes) and 9 (5 minutes), and umbilical artery pH was 7.340. The placenta did not separate naturally 15 minutes after birth. Hysterectomy was conducted without manual removal of the placenta. Operating time was 2 hours 3 minutes and total blood loss amounted to 1412 g. The patient recovered smoothly after surgery.

In examining the removed uterus, the placenta was low‐lying but placenta previa was not present. The cervix was normal in appearance. Most of the placenta could be easily separated. Pathological examination revealed an adherent placenta in a small portion of the lower posterior uterine corpus wall without placental invasion. We concluded that the marginal sinus of the placenta descended through the cervix to the external os like funneling of the amniotic membrane in cases of threatened preterm delivery.

## 3 DISCUSSION

To obtain a general “big picture” image, MRI is superior. In this case, however, transvaginal ultrasonography combined with color imaging proved to be more accurate for observing details of the cervical region. The marginal placental sinus descended through the cervix like amniotic membrane funneling observed in cases of threatened preterm delivery.

As this is the first case of its kind, we were not able to confirm the accuracy of ultrasonography. MRI indicated the possibility of invasive placentation into the cervix. The placenta did not separate naturally 15 minutes after the baby's birth. As the woman and her family did not desire to preserve her fertility, a hysterectomy was conducted.

However, the marginal sinus did not invade to the uterine wall, and no direct blood flow connection was found between the descended marginal sinus and the intracervical vessels. The placenta could have been separated if manual removal had been tried. Looking back at this case, cesarean section was required to avoid sinus rupture; however, hysterectomy could have been avoided if we had confirmed the accuracy of ultrasonography.

Several decades ago, Ferguson^1^ reported that marginal sinus rupture was the most common cause of external hemorrhages in late pregnancy. The same author,^2^ however, commented in another report that many clinicians doubted the existence of marginal sinus rupture. Justifying these doubts, until now, no publication has reported marginal sinus rupture since the 1970s. The concept of marginal sinus rupture seemed to disappear. The marginal sinus itself is not an abnormal structure. Moreover, blood escape after delivery makes the sinus collapse, which does not allow specific findings of the delivered placenta by macroscopic or microscopic examinations. This might create doubts regarding the existence of marginal sinus rupture.

We hypothesize that rupture of the marginal sinus of the placenta would have happened if there was a vaginal delivery in this case. Ohira et al^3^ reported that the presence of the marginal sinus was associated with subsequent cesarean delivery because of antepartum vaginal bleeding in cases of low‐lying placenta. Taga et al^4^ also reported that women with low‐lying placenta required emergent cesarean delivery because of bleeding in the presence of the marginal sinus. We thought that rupture of the marginal sinus of the placenta exists.

Ultrasound is currently the gold standard for identifying low‐lying placentation. When the placental edge relocates near or reaches the internal os in the third trimester, low‐lying placenta or placenta previa is diagnosed.^5^ However, whether the definition of the placental edge is the parenchyma or the placental marginal sinus is unclear.

Sometimes with low‐lying thick placenta, we see contact between the placental fetal surface and the internal os even though the maternal side of the placenta does not cover the internal os. This cannot be diagnosed as placenta previa. The marginal sinus has contact but no direct connection to the uterine wall. Noting these similarities, the marginal sinus only reaching to the internal os cannot be diagnosed as placenta previa. From a pathological viewpoint, the definition of the placental edge in diagnostic imaging should be the parenchyma. However, from a clinical viewpoint, when the marginal sinus reaches the internal os, planned cesarean section should be conducted to avoid bleeding. In summary, the marginal sinus reaching the internal os is not pathologically placenta previa, but should clinically be treated as placenta previa. Ishibashi et al^6^ proposed marginal sinus placenta previa as a mild type of placenta previa.

## 4 CONCLUSION

This is the first reported case of descent of the placental marginal sinus into the external os. From this case, we believe that marginal sinus rupture does exist. Furthermore, from a pathological viewpoint, the definition of the placental edge in diagnostic imaging should be the parenchyma. Clinically, however, when the marginal sinus reaches the internal os, it should be treated as placenta previa.

## CONFLICT OF INTEREST

None declared.

## AUTHOR CONTRIBUTION

ER: wrote the manuscript. SN: helped in identifying appropriate images. IN, RK, KY, and TY: involved in patient management. All authors reviewed the manuscript.
